# Animal models of post-ischemic forced use rehabilitation: methods, considerations, and limitations

**DOI:** 10.1186/2040-7378-5-2

**Published:** 2013-01-23

**Authors:** Jessica M Livingston-Thomas, R Andrew Tasker

**Affiliations:** 1 Department of Biomedical Sciences University of Prince Edward Island, 550 University Avenue, Charlottetown, PEI C1A4P3, Canada

## Abstract

Many survivors of stroke experience arm impairments, which can severely impact their quality of life. Forcing use of the impaired arm appears to improve functional recovery in post-stroke hemiplegic patients, however the mechanisms underlying improved recovery remain unclear. Animal models of post-stroke rehabilitation could prove critical to investigating such mechanisms, however modeling forced use in animals has proven challenging. Potential problems associated with reported experimental models include variability between stroke methods, rehabilitation paradigms, and reported outcome measures. Herein, we provide an overview of commonly used stroke models, including advantages and disadvantages of each with respect to studying rehabilitation. We then review various forced use rehabilitation paradigms, and highlight potential difficulties and translational problems. Lastly, we discuss the variety of functional outcome measures described by experimental researchers. To conclude, we outline ongoing challenges faced by researchers, and the importance of translational communication. Many stroke patients rely critically on rehabilitation of post-stroke impairments, and continued effort toward progression of rehabilitative techniques is warranted to ensure best possible treatment of the devastating effects of stroke.

## Introduction

In 1973, Norwegian neuroscientist Alf Brodal wrote: “since regeneration of transected central axons has never been convincingly demonstrated in higher mammals, it seems in most instances that one must resort to the assumption that intact fibres ‘take over’ for the damaged ones” [1]. Although this portrays part of the story, decades later we now know that there are a number of processes involved in post-injury neuroplasticity, causing a significant shift in the way we think about neurorehabilitation. As research into the complicated phenomenon of functional recovery and use-dependent reorganization in the brain continues, the importance of valid animal models of post-stroke rehabilitation has become clear.

Almost 850,000 North Americans experience a stroke each year [2,3]. In addition to a high mortality rate, the majority of those who survive a stroke are left with motor disabilities [3], such as upper extremity impairments. Many patients remain chronically impaired in the months and years following a stroke, which vastly impacts quality of life and contributes to post-stroke depression [4]. With an aging population, a drastic increase in the societal burden of stroke can be expected if progressive improvements in stroke treatment and post-stroke rehabilitation are not made.

While preventive therapies and lifestyle modifications are known to reduce the incidence of stroke and associated mortality [5], the only effective drug presently available to treat stroke is tissue plasminogen activator (tPA). TPA works only in the event of ischemic stroke, by dissolving the offending blood clot to restore blood flow and prevent further damage. However, it is effective only in a fraction of stroke patients [6] due in part to a narrow therapeutic time window [7,8]. Neuroprotective drugs are effective in experimental settings, but to date, none have proven clinically effective [9]. Hence, continued effort toward progression of rehabilitative techniques and enhanced communication between clinical and experimental researchers is warranted to ensure best possible care and recovery of patients who suffer the devastating effects of a stroke.

### Constraint induced movement therapy

**Figure 1:**
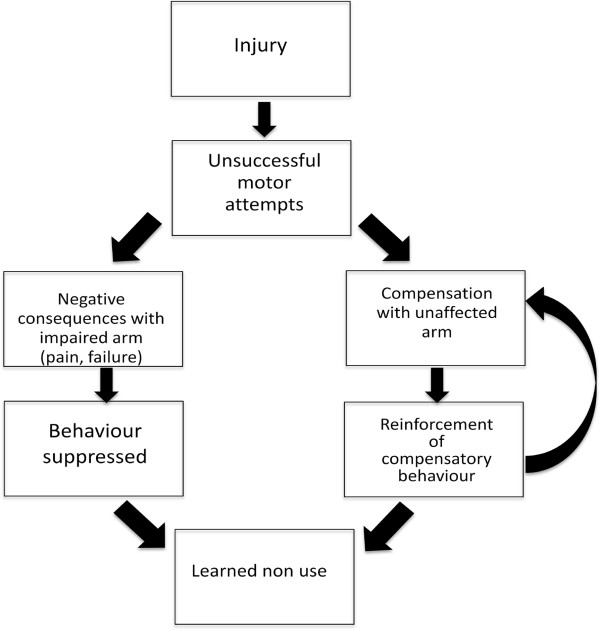
**Following injury to the forelimb, attempts to use the impaired limb lead to unsuccessful motor attempts.** The consequences include pain and failure, creating negative feedback which leads to suppression of the behaviour. Meanwhile, the unaffected or less affected forelimb is used with higher success, leading to reinforced compensation. Together, these constitute the phenomenon of learned non-use. Adapted from Taub et al. [11].

One rehabilitative technique that has been developed to increase use and improve function of the upper limb in survivors of stroke is constraint induced movement therapy (CIMT) [10]. The therapy discourages ‘learned non-use’ , first described by Taub et al. [11]. Learned non-use is a phenomenon whereby movement is initially suppressed due to failure and adverse consequences encountered when a subject attempts to use the affected limb. This results in persistent compensatory behaviours and subsequent suppression of use of the impaired limb, even when function may eventually be possible (Figure 1). Through constraint of the unaffected limb and subsequent forced use of the affected one, CIMT encourages positive feedback about the limb’s functional potential. Generally, the constraint device is worn for most waking hours during a two week period [12,13], and is accompanied by intensive repetitive task practice (RTP) performed daily using the (unconstrained) impaired limb. RTP is performed in conjunction with shaping, during which participants engage in meaningful functional activities with measurable progressions for which they receive positive feedback as the activities become increasingly difficult. In addition to shaping, other behavioural techniques such as home practice and problem solving sessions are used to aid in the transfer of functional gains to performance of daily activities [14].

CIMT has been clinically shown to improve functional outcome [10,12,13,15,16], even when administered to patients with chronic deficits [17], however, the therapy presents several obstacles. CIMT is expensive due to the intensive therapy requiring specialized professionals. Moreover, despite the fact that subjects are required to sign a ‘behavioural contract’ [15,18], designed to increase incentive and ensure motivation, CIMT is a demanding therapy for stroke patients who have diminished physical capacity. This raises concerns about compliance issues [19], and several modifications of the therapy have been investigated in an attempt to reduce the intensity of the therapy [20-22]. While results of these studies are promising, larger confirmatory research is required. Further, the procedure has been developed for and tested in patients that retain some level of function, according to a general inclusion/exclusion criteria that include the ability to initiate finger movements [15], and extension movement of at least 20 degrees in the wrist and fingers [13,17,22]. This limits the generalization of study results to all stroke patients that are hemiparetic [23,24].

### Experimental rehabilitation in animals: opportunities and challenges

Valid experimental models are essential for the development of rehabilitative therapies to treat survivors of stroke. Mechanisms underlying functional recovery exist at cellular, circuit, and system levels, and should be examined at each. By modeling CIMT in animals, it may be possible to elucidate underlying processes responsible for functional benefits, and eventually manipulate these processes to accelerate or enhance post-stroke recovery. Indeed, there have been several attempts at the development of animal models of rehabilitation that resemble CIMT [25-30]. These have provided important insights into the numerous variables that need to be considered, such as 1) the choice of an appropriate animal model of stroke, 2) development of rehabilitation models that best model particular and appropriate aspects of the regimen, and 3) appropriate identification and interpretation of outcome measures. This review provides an overview of these variables, and addresses considerations and limitations of each.

### Animal models of ischemic stroke

In-depth investigation into the complicated processes underlying functional recovery relies critically on the use of live whole animals. A variety of animal models including pigs [31] and non-human primates [32] have been explored, but rat models of stroke are by far the most common choice for researchers. This is largely due to the similar neurovascular branching to humans [33,34], relatively low cost [35], and high number of validated behavioural tests of functional outcome in the rat [33,35,36]. In the case of forelimb rehabilitation studies, rats are especially valued for the similarities to the limb movements of the human arm [37].

When choosing a rat model of stroke, further consideration must be given to inter-strain differences. Experimental stroke performed identically in different strains of rats can produce markedly different damage [38], and each can respond differently to post-stroke treatments [39]. With several strains to choose from (e.g. Sprague Dawley, Wistar, Long Evans), it is important to optimize the chosen model to a chosen strain. Ultimately, the choice is usually a matter of personal preference, and several strains are acceptable, however caution should to be applied when interpreting results obtained using one strain relative to other studies using other strains.

A further consideration is the age of the animals to be used in the study. Although clinical stroke occurs largely in the elderly, using aged animals in experimental settings presents challenges. Increased post-surgical mortalities [40,41], the need for more intensive post-operative care [42], and the greater investment of resources required to age the animals make them less than ideal subjects in basic research. For these reasons, the majority of post-stroke studies are done on young adult animals (~4 months old). There is increasing criticism of this practice, however, as some argue that the use of young animals is less clinically relevant, and may contribute to issues with translating research findings [9,40,43,44].

#### Experimental stroke models

Stroke is an extremely diverse condition, and there is no ideal experimental stroke model. Rather, it is important to consider how well a particular model will address a specific experimental question. Criteria to consider include pathophysiological processes, lesion size and reproducibility, and physiological variables that can be monitored and maintained.

All experimental stroke models are associated with some variability in lesion size and location, especially important parameters when studying rehabilitation. Because recovery of function is largely attributed to numerous neuroplastic changes on a cellular level [45-47], it is important to ensure sufficient unaffected or salvageable tissue remains to act as a substrate for recovery processes. Furthermore, some stroke model complications can have a direct impact on behavioural outcome. For example, poor overall health due to loss of body weight, stress from surgery, infections, and collateral damage to a non-targeted brain area (e.g. those associated with cognitive function) can all impair functional performance. These factors must be considered when choosing a stroke model for evaluation of rehabilitation.

#### Middle cerebral artery occlusion by intraluminal filament

The middle cerebral artery (MCA) is often affected in ischemic stroke, hence the development of the most commonly used animal stroke model: middle cerebral artery occlusion (MCAo). This approach aims to cause ischemia to the targeted area of the brain by occluding this downstream major vessel. The MCA can be accessed via the internal [48] or external [49] carotid artery, and occluded temporarily [49] or permanently [50]. Transient MCAo is achieved by temporary insertion of a filament into the MCA, which is later removed resulting in blood flow restoration. Permanent MCAo involves leaving the filament in the MCA, or using a clip to permanently occlude blood flow.

MCAo does not require an invasive craniectomy, as arteries are accessed at a midline ventral incision point. Infarcts often comprise both cortical and striatal damage, a common pathology following stroke [51]. However there can be non-targeted damage to brain regions outside the vascular territory of the MCA [52], and associated unintended behavioural impairments [45]. During external carotid artery occlusion, facial muscles may be affected by ischemia, leading to feeding problems, weight loss, and mortality. Furthermore, even subtle variations in surgical technique can have an impact on outcome, largely contributing to the variability inherent in the model [53].

#### Endothelin-1

Endothelin-1 (ET-1) is a potent vasoconstrictor that can be used to induce ischemia directly when applied topically [54,55] or injected intracerebrally [56,57] or indirectly if injected proximal to the MCA [58]. ET-1 affects arteries in the immediate area of injection, and causes a rapid and temporary reduction in blood flow followed by reperfusion over several hours [33], making it more representative of the clinical condition compared to the immediate reduction and reperfusion associated with MCAo [59]. An advantage of this model is the ability to ‘fine tune’ infarcts by adjusting the concentration, volume, and stereotaxic placement of the microinjections. This even allows researchers to model white matter damage, thought to be relevant to lacunar infarctions observed in many stroke patients [60]. Infarcts achieved using this model can be more localized than those resulting from traditional MCAo, and have been shown to result in long-lasting behavioural deficits [43,56,57]. However, the exact mechanism of vessel occlusion is not well characterized, and occlusion duration is not easily controlled.

#### Photothrombosis

Photothrombotic stroke models induce cortical damage by the systemic injection of a photoactive dye (e.g. Rose Bengal) followed by irradiation by a light beam [61]. This method can be used to produce widespread damage by targeting the middle cerebral artery [62,63], or to produce more localized lesions by application to the vasculature directly in the targeted brain area [29,61,64,65]. This results in the generation of free radicals, leading to focal damage, platelet activation, and aggregation in vessels within the irradiated area, resulting in ischemia.

Photothrombosis allows the experimenter to easily target precise regions of irradiation, and results in relatively reproducible lesions. This method is associated with a low mortality rate and consistent infarcts with precise location and size [64]. Because the photoactivation occurs through a thinned area of skull, craniectomy is not necessary. However, because the model is based on photoirradiation through the skull onto the brain surface, the resulting infarct is only cortical. Furthermore, this model does not produce a penumbral region that resembles that observed in clinical stroke [66].

#### Devascularization

Devascularization of the cortex can be achieved by pial stripping, a process whereby surface vasculature is physically removed [67-69], for example by rubbing. The resulting damage can extend to white matter that lies beneath the devascularized area. Though relatively well-controlled, there can be mechanical damage to surrounding tissue and vessels, resulting in hemorrhage [59]. Like photothrombosis, this method does not permit reperfusion [59].

#### Other stroke models

The stroke models described above are those most commonly used to study post-stroke rehabilitation paradigms (see Table 1 for summary). There are several other models of focal ischemia that are widely used for other aspects of stroke research such as embolization and spontaneous infarction using hypertensive rats [34], as well as of multi-focal, global ischemia, and hemorrhagic models (for comprehensive reviews, see [59,70]).

**Table 1:** Common stroke models used in studies of rehabilitation

**Stroke model**	**Advantages**	**Disadvantages**	**References**
MCAo (indirect ischemia)	+Models transient or permanent ischemia;	-Large and variable infarcts;	[48,49,51,52,71]
	+No craniectomy required;	-Collateral damage due to non-targeted vasculature;	
	+Results in cortical and striatal damage	-Feeding problems may occur;	
	+Widely used and well-characterized	-Some mortality	
Endothelin-1 (indirect or direct ischemia)	+Models transient ischemia;	-Requires removal of some skull tissue;	[55-58,72]
	+Can produce cortical and striatal damage;	-Less control over duration of occlusion;	
	+Ability to control precise variables (e.g. concentration, injection volume, stereotaxic coordinates) resulting in localized lesions;	-Mechanism of vessel occlusion not well elucidated	
	+Can be used to model lacunar infarcts		
	+Low mortality rate		
Photothrombosis (indirect or direct ischemia)	+Models permanent ischemia; low mortality rate;	-Requires skull thinning (direct);	[61,63-66]
	+Precise control over lesion size and location (direct);	-Can only produce cortical damage (direct);	
	+Full craniectomy is avoided	-Collateral damage to non-targeted areas (indirect)	
		-No penumbra	
Devascularization (direct ischemia)	+Models permanent ischemia;	-Requires removal of skull tissue;	[59,67-69]
	+Relatively good control over lesion size location;	-Mechanical damage can occur to surrounding tissue and vessels;	
		-Can produce surface damage only;	
		-No penumbra	

A summary of the advantages and disadvantages of several commonly used models of experimental stroke.

### Choosing a model of rehabilitation

Over the past two decades several creative animal models of forced use therapies have been developed, however the major challenge researchers face is that of subject motivation. As highlighted above, CIMT involves constraint of the nonparetic limb for most waking hours, which forces use of the impaired limb for daily tasks as well as intensive therapist-led exercises. In rats, this presents a challenge. Certainly, the affected forelimb can be constrained [25-27], however increased animal stress [57,73,74] and lack of behavioural pressure to rely on the impaired forelimb [27,57] can be problematic. Some researchers have opted to shift focus away from constraint *per se* and toward forced use. Many also incorporate task-specific exercises to model the RTP associated with CIMT. However, this results in the need to ‘trade off’ aspects of the rehabilitation, in an attempt to replicate specific components of therapy. For example, experimental rehabilitation may require unconstrained bilateral (rather than unilateral) forced use, or less-intense voluntary paradigms to reduce animal stress and increase behavioural incentive. As noted by Taub and Uswatte [18], the ‘constraint’ used in CIMT is intended as a means to induce patients to use the affected extremity for a large proportion of the time and for a variety of activities, thus other strategies that induce similar pressures are likely to result in similar use-dependent reorganization and functional benefit. Some of the more common techniques used in animals are summarized in Table 2 and described below.

**Table 2:** Models of experimental forelimb rehabilitation

**Rehab model**	**Advantages**	**Disadvantages**	**References**
Constraint	+Most direct model of CIMT;	-Constraint devices may be stressful, confounding results;	[25,27,75]
	+Allows constraint for specific durations thereby allowing the evaluation of various durations of therapy;	-Lack of behavioural pressure to use paretic arm despite constraint	
	+Conducive to studies of unilateral forced use		
Forcing use with locomotion	+Stimulates use of the paretic limb in a less aversive paradigm	-Can be stressful (involuntary forced use);	[27,57,76-81]
		-May lack control over intensity (voluntary forced use);	
Encouraging use	+Stimulates use of the paretic limb in a less aversive paradigm	-Complicated by other non-forced use therapy components such as cognitive stimulation;	[82-86]
		-Usually involves bilateral forced use	
Task specific exercises	+An addition to rehabilitation that models task specific shaping exercises of CIMT	-Requires the desire of animals to participate in a demanding task	[27,76,77,82-84,86]

Summary of advantages and disadvantages of previously described animal models of rehabilitation.

#### Constraint

The principle behind this strategy is straightforward: constrain use of the unaffected forelimb, thereby forcing the animal to use the affected forelimb. Debow et al. [27] describe employing a sleeveless jacket, wrapped around the upper torso of the rat and attached to a metal wrist bracelet. A major benefit of this paradigm is that the duration of the restraint is easily controlled; i.e. the bracelet was clipped to the jacket for 8 hours/day. The disadvantage is that the continuous wearing of the jacket and the complete restriction of the unaffected forelimb for most of the day could exacerbate animal stress.

A more permanent paradigm of constraint is plaster casting, whereby following stroke surgery, the affected forelimb is plastered to the torso [25,26,75]. Like the constraint jacket, this could result in animal stress and ultimately confound results, and these paradigms alone may not create the behavioural pressure required to intensely force use of the impaired forelimb.

#### Forcing use by locomotion

Some researchers have focused directly on forcing use of the impaired limb, exploiting the fact that in order to run or walk, animals need to engage the impaired forelimb to some degree. The intensity of such therapies varies from 10–60 minutes of locomotion (and from < 1 km to 7 kms per day) and lasts 3–5 weeks [25,27,57,76-81]. This form of rehabilitation can be involuntary, using motorized running wheels or treadmills [44,74,76,78-80,87], or voluntary, by allowing free access to running wheels [77,81,87] or pet activity balls [57].

In the voluntary models, the use of the impaired forelimb may still be considered ‘forced’ because of the necessity to engage the impaired forelimb to perform the movement, while the appetitive nature of the activity is believed to reduce the stress experienced by the animal [74]. Because movement is voluntary, the experimenter has less control over rehabilitation intensity, however the resulting self-inflicted intensity could be regarded as an additional measure of performance [57,81]. There is some evidence that a voluntary rehabilitation paradigm may exert a more beneficial effect than involuntary [74].

#### Encouraging use with enriched environment

In 1947, neuroscientist Donald Hebb noted that laboratory rats that had been taken home as pets had better learning and problem-solving skills that those who lived in standard laboratory conditions [88]. This prompted research into enriched environments (EEs) which have since been found to enhance sensory, cognitive, and social stimulation, as well as improved recovery following stroke [82-85,89].

Exposure of rats to enriched environments provides ample opportunity for engagement in voluntary nonspecific activity, as a major component of these environments is the presence of novel objects and climbing apparatuses. As such, they generally encourage voluntary movement in a way that stimulates use of the impaired forelimb. While the contribution of the motor enrichment component of EE to functional recovery has yet to be elucidated, this environment represents a basic voluntary use paradigm that has shown significant positive results when scrutinized by systematic review [89].

#### Task specific exercises

**Figure 2:**
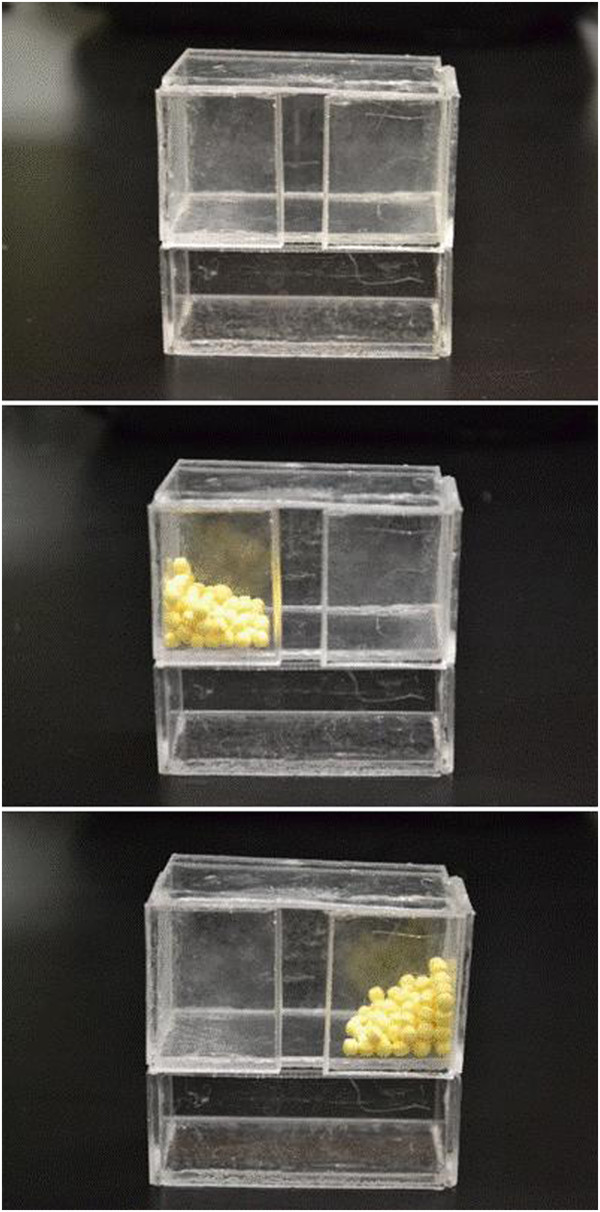
**A task-specific reaching exercise can be administered using an apparatus such as this.** By piling palatable sugar pellets to one side of the box (opposite from the impaired forelimb) rats are encouraged to use the impaired limb to reach through the center slot. The intensity can be altered by controlling the accessing time, number of pellets, and height of the reaching box.

CIMT includes RTP exercises that involve grasping, gripping, and manipulating objects [12,13], therefore many experimental researchers combine a form of rehabilitation described above with a task specific exercise [27,57,76,77,82,83,86,90], [91]. Experimentally, these RTP exercises are often modeled using pellet reaching tasks. Animals are provided palatable sucrose pellets that require reaching with the impaired limb. This can be achieved by placing pellets on the appropriate side of a tray, shelf, or well [57,77,83,90] (Figure 2), or by using a modified staircase apparatus (described in *Reaching Tests*) wherein the descending stairs have been replaced on the impaired side with a pile of pellets [82,84,86].

#### Initiating rehabilitation

While CIMT is generally initiated as soon as the patient is medically stable, it is effective when initiated even several years post-insult [17,21]. However, some animal studies have shown that early initiation of rehabilitation can result in larger infarcts and worse functional outcome [28,92]. Therefore, it is recommended that therapy be delayed until several days post-infarction. Unlike humans, rats also exhibit spontaneous recovery on many functional tests in a relatively short time frame; delaying rehabilitation too long may result in missed opportunity to accelerate that recovery. Generally, experimental rehabilitation is initiated between post surgical day 3 and 7 [27,57,76,77,83,85,86].

The time course of post-ischemic plasticity is interconnected with the cascade of events that occur on a cellular level in the brain following stroke. The surviving tissue constitutes a growth promoting microenvironment [45,47,93,94] wherein the expression of survival, repair, and plasticity genes are temporally expressed [94]. In the first days following ischemia, there is resolution of edema and diaschisis, followed by altered expression of growth-stimulating and -inhibitory genes. Further, a diverse immunological response is initiated, the role of which remains controversial. Inflammation and activation of microglial and other immunological cell types is believed to have both harmful [95] and beneficial roles [95,96].

Considering that all post-ischemic processes are dynamic and dependent on the severity of the initial insult, the precise temporal and physical properties of these recovery mechanisms vary.

### Choosing functional outcome measures

When evaluating post-stroke deficits and subsequent recovery in an animal model, it is essential to identify functional assessments that have translational relevance to the clinic. Several considerations that must be made when choosing behavioural tests include the sensitivity of the test to the damage incurred, the time required to pre-train and test animals, and the possible confounding properties of the tests and testing schedule chosen. The latter is especially important in studies of rehabilitation, in which the tests themselves can affect post-stroke neuroplasticity [59].

Animal behavioural testing can be extremely time consuming, and requires an experienced researcher to administer. Animals must be pre-trained on most tests, sometimes for periods of several weeks. This reduces the confounding effects of stress and fear on behaviour, and ensures collection of reliable data. Following training, it is important that animals are tested in an experimenter-blind fashion to avoid test interpretation bias. Administration time varies greatly between tests, and analysis can be laborious. Many forelimb function assessments involve filming the test for later detailed video analysis. Together with the fact that experimental animals are usually kept on a strict light:dark schedule, these factors all affect the logistical feasibility of any experimental study, often requiring multiple cohorts to achieve the desired number of replicates.

Several behavioural tests of forelimb function have been characterized in rat stroke models; the most commonly used are described below (see Table 3 for summary). Each test is sensitive to measuring deficits associated with a particular area of damage, therefore it is useful to employ a battery of tests to capture various aspects of damage and recovery [97]. Simpler tests of gross motor function categorized by ordinal scoring scales are often employed as post-surgical screening tools, to ensure surgical success and to stratify animals into groups based on initial post-stroke deficit [76,87]. Because it is important that the animals have been acclimated to the experimenter performing the tests, when possible, one experimenter should administer the test on all testing days. If this is not feasible then steps should be taken to ensure inter-experimenter reliability. Prior to experimental stroke, it is useful to obtain baseline performance by pre-testing.

**Table 3:** Commonly employed behavioural tests of forelimb function

**Behavioural test**	**Purpose**	**Advantages**	**Disadvantages**
NSS	Awards an overall score for determining general deficit	+Encompasses a range of assessments, then compiles them into a single measure	-Time intensive;
			-Does not inform about the nature of specific deficits;
Cylinder test	Assesses spontaneous forelimb use	+Fast and easy to administer;	-Video analysis can be time intensive
		+Allows for analysis of a number of functional movements	
Montoya staircase test	Assesses forelimb extension, dexterity, side bias, independent use of forelimbs	+Easy to administer;	-Intensive tests training which requires food deprivation;
			-May confound results of task-
		+Allows for analysis of both reaching distance and forepaw dexterity	specific rehabilitation if performed often
Single pellet reaching task	Assesses forelimb dexterity	+Allows for in-depth analysis of the animal’s performance by isolating a single reach attempt	-Intensive tests training which requires food deprivation;
			-May confound results of task-specific rehabilitation if performed often
Horizontal ladder test	Assesses forelimb stepping, placing, and coordination during locomotion	+Can assess forelimb and hind limb damage	-Can be complicated by post-surgical immobility
Forelimb flexion test	Assesses postural reflex	+Fast and easy to administer	-Measures only postural reflexive position,
			-Only awards a 0–2 score.
Forelimb placing test	Assesses forelimb function and placing deficits	+Fast and easy to administer	-Measures only reflexive sensorimotor response;
			-Can be difficult to distinguish between reflexive response and initiated movement, therefore experimenter must be experienced at determining validity

A summary of advantages and disadvantages of various tests of functional deficit and recovery.

#### Neurological severity score

The neurological severity score (NSS) is a broad measure of functional ability following experimental stroke. The NSS is compiled from scores on up to 20 tests measuring reflexes, balance, sensory responses, and motor functions [49,75,79]. A list of tests and observations are used to assign a score (maximum score indicates severe deficits) that encompasses animals’ posture, startle reflex, circling, and more (see Table 4).

**Table 4:** Generalized neurological severity score

***Motor tests (maximum 6)***	
*Raising rat by tail (maximum 3)*	
Flexion of forelimb	1
Flexion of hindlimb	1
Head moved >10° to vertical axis within 30 s	1
*Placing rat on floor (maximum 3)*	
Normal walk	0
Inability to walk straight	1
Circling toward paretic side	2
Falls down to paretic side	3
***Sensory tests (maximum 2)***	
Placing test (visual and tactile test)	1
Proprioceptive test (pushing paw against table edge)	1
***Beam balance tests (maximum 6)***	
Balances with steady posture	0
Grasps side of beam	1
Hugs beam and 1 limb falls down from beam	2
Hugs beam and 2 limbs fall down from beam, or spins on beam (60 s)	3
Attempts to balance on beam but falls off (40 s)	4
Attempts to balance on beam but falls off (20 s)	5
Falls off; no attempt to balance or hang on to beam (20 s)	6
***Reflex absence and abnormal movements (maximum 4)***	
Pinna reflex (head shake when auditory meatus is touched)	1
Corneal reflex (eye blink when cornea is lightly touched with cotton)	1
Startle reflex (motor response to snapping a clipboard )	1
Seizures, myoclonus, myodystony	1
***Total maximum points***	**18**
*Final score: 13–18= severe injury; 7–12= moderate injury; 1–6= mild injury.*	

A general summary of the evaluations commonly used to assess neurological severity in experimental models. This table can be modified to contain particular tests that are pertinent to the experimental design being used.

#### Cylinder test

The cylinder test is widely used to evaluate spontaneous forelimb use during exploratory behaviour [26,27,43,56,65,82,84,85], [98,99]. First described by Schallert et al. [100], this test is based on the observation that rats explore vertical surfaces by lifting and bracing themselves with their forequarters. An intact animal typically uses both forelimbs equally for support, however following unilateral lesion animals rely more heavily on the unimpaired forelimb.

**Figure 3:**
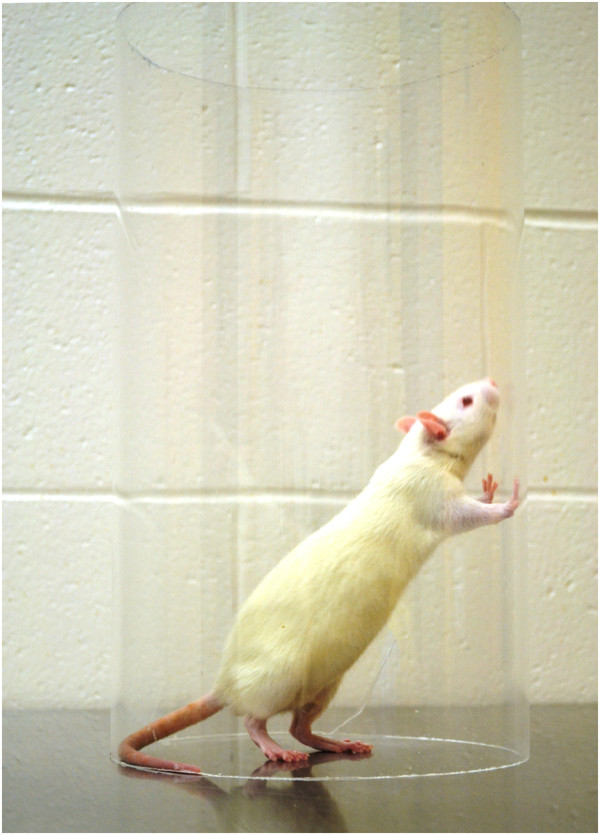
**The Schallert cylinder test **[100]** is performed by placing the rat into a clear plastic cylinder and analysing the proportion of contralesional limb use during exploring and rearing activities.**

To perform the test, the animal is placed into a transparent plastic cylinder which encourages vertical exploration (Figure 3). The animal is then video recorded while rearing and exploring, generally for five to ten minutes or for a predetermined number of rears. The ratio of wall placements observed for the intact and impaired limb is determined by slow motion video analysis, and used to calculate the percent usage of the impaired forelimb. Multiple camera angles and mirrors can be used to obtain additional measurements, such as use of limbs during weight bearing and landing. This test requires minimal training (1–2 exposures), is easy to administer, and takes a moderate amount of time to analyse.

#### Reaching tests

**Figure 4:**
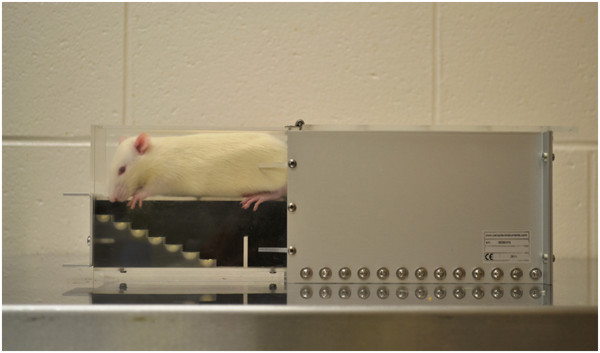
**The Montoya staircase test **[101]** measures dexterity and sensorimotor function by analysing pellet reaching behaviour.** Rats are placed into apparatus with seven descending steps on each side. Steps are baited with sucrose pellets to encourage reaching.

The Montoya staircase test [101] is used to assess forelimb sensorimotor capacity, dexterity, and coordination [27,57,76,78,82,84-86]. Animals are placed into an apparatus consisting of an elevated platform with seven descending steps on either side (Figure 4). Each step is baited with multiple palatable sucrose pellets, at progressively deeper levels. Animals are generally given 15 minutes to attempt to reach all pellets. In order to consume pellets they must make a successful dexterous movement requiring motor function and sensory feedback. Based on the number and position of remaining pellets at the conclusion of the 15 minute interval, the staircase test allows for bilateral measurement of forelimb extension and grasping skills. Pre-training on this test is extensive (2 weeks) and usually requires modest food deprivation, however the time for administration and analysis of the results is minor.

An alternative test used to assess forelimb function is the single pellet reaching task. In this test, animals are presented with a single pellet at a time, which must be accessed by reaching through a slot [77,86], sometimes at increasingly difficult distances [90]. This test allows for more in-depth analysis of the animal’s performance, as reaching attempts (as well as successes) can be recorded. Accuracy can be determined using the equation: (number of successful reaches)/(total number of reaching attempts) × 100. Video analysis can be done to perform more in-depth kinematic analyses.

#### Horizontal ladder test

The horizontal ladder test is used to assess skilled locomotor movements including stepping, placing, and inter-limb coordination [27,43,57,78,83,84,86]. First described by Metz & Wishaw [67], it offers comprehensive qualitative and quantitative analysis of post-stroke condition.

**Figure 5:**
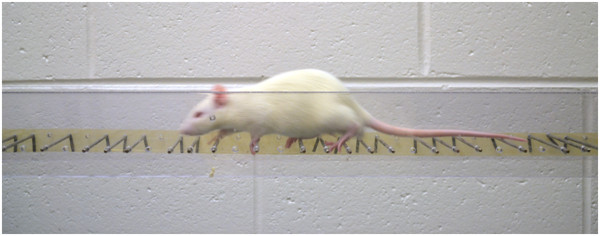
**The horizontal ladder test is used to assess forelimb function when crossing a platform of unevenly spaced rungs.** The number of foot slips is determined by video analysis.

The apparatus is a horizontally positioned ladder which the animal crosses to escape aversive stimuli (noise and light) (Figure 5). The spacing between the rungs of the ladder is variable and can be changed to prevent the animal from learning either the absolute or relative location of the rungs. The animal is video recorded while crossing the apparatus, to determine the number of foot slips made by each limb. Training on this test is minimal, while analysis can be moderately time intensive due to the video analysis required.

#### Forelimb flexion test

The forelimb flexion test was developed by Bederson et al. [102] to examine upper body posture while the animal is suspended by the tail. The test involves suspending a rat above the home cage or a table top and observing the position of the impaired forelimb. Intact animals extend both forelimbs directly toward the surface beneath, while post-stroke animals flex the contralateral forelimb (at a 45–180 degree angle to the body). Depending on stroke severity, this is sometimes accompanied by torso twisting. Posture is normally assigned a score from 0–2, wherein 0 = no flexion, 1 = flexion of the forelimb, and 2 = forelimb flexion with twisting of the torso. This test requires no pre-training, and is very fast to administer.

#### Forelimb placing tests

**Figure 6:**
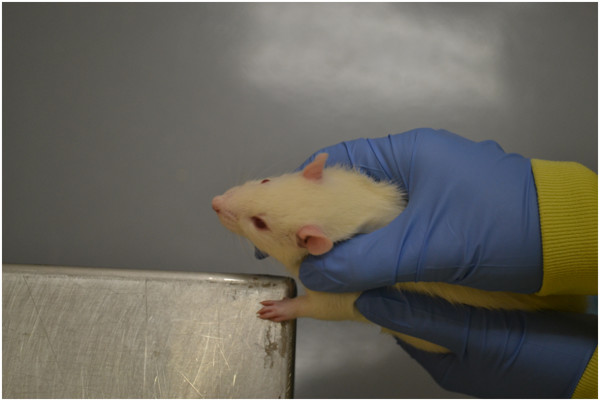
**Forelimb placing tests involves eliciting a response from either tactile or vibrissae stimulation.** Three limbs are held secure while the remaining one is tested for the ability to place the forelimb on the table edge in response to stimulation.

The forelimb placing tests are used to examine sensorimotor integration in response to tactile and proprioceptive stimuli [103]. To test forelimb function, a rat is held by the torso with 3 limbs secured and the limb to be tested hanging freely. The vibrissae or wrist of the animal is brushed against the edge of a table several times to test vibrissae- and tactile-stimulated forelimb placing, respectively. In intact animals, this stimulation elicits the response of placing the forelimb being stimulated on the table (Figure 6). Neurological damage from experimental stroke causes this response to be impaired. This test requires approximately 4–5 pre-exposures for acclimation, and administration of this test is fast.

## Discussion

With an increasingly aging population, high incidence of stroke, and few effective medical interventions, it is vital to gain a better understanding of the mechanisms underlying functional recovery in order to refine existing rehabilitative therapies and to develop new techniques. In order to understand the mechanisms underlying rehabilitation, animal models are of critical importance. Addressing all aspects of human stroke using any given animal model will never be possible, but many have proven indispensible for contributing to our understanding of the mechanisms underlying ischemic and neuroplastic processes.

While animal models remain critical to basic stroke research, there are limitations in the translation of research findings to clinical practice. As with other experimental conditions, stroke models represent a simplified version of an extremely complex human condition. Gender, age, pre-stroke condition, stroke severity, and co morbidities are highly variable in the clinical population, but tightly controlled in experimental studies. The amount of damage and subsequent severity of impairment are also disjointed between the animal and human population, as animals both require and can survive more severe neurological damage in order to produce relevant functional deficits. Furthermore, inherent complications of stroke models outlined must be appreciated.

With the plethora of variables to consider at each step when examining post-stroke rehabilitation, modeling rehabilitation presents a major challenge and remains fundamentally different from the clinic. Stroke patients undergoing CIMT receive supervised, assisted, and highly motivated rehabilitation with trained experts. Experimental rehabilitation is largely hands-off, and animals are more difficult to motivate. Decisions must be made to best represent the validity of a model with respect to a particular aspect of the therapy. Such decisions may result in compromises in the intensity of the therapy, or the use of bilateral rather than unilateral stimulation, in order to address stress and behavioural pressure. However, cortical reorganization and functional benefit may still share underlying processes to those responsible for the benefits of CIMT. In the clinic, constraint is considered the tool that induces the beneficial use of the impaired limb and discourages learned non-use [18].

As is the case in clinical evaluations, efficacy of rehabilitation depends on the use of functional measures. In order to accurately assess a model of rehabilitation, it is important that researchers choose appropriate outcome measures that will be sensitive both to the nature of the damage caused by the stroke model, and to possible improvements that could be attributed to the therapy. Furthermore, tests of gross motor movement (e.g. forelimb placing) and those that use ordinal scales (e.g. forelimb postural reflex) can result in a ‘ceiling effect’ , wherein subjects’ performance scores return to normal levels while some impairment remains undetected. This is a challenge faced by clinical researchers, who use scaled outcome measures like the Barthal index (BI), motor activity log (MAL), and functional independence measure (FIM), also susceptible to the ceiling effect [21,104-106]. Thus, it is important that a researcher chooses a battery of tests that encompass a variety of movement types. Importantly, behavioural outcome is a culmination of a number of processes that can be affected by various aspects, including recovery, compensatory strategies, stress, anxiety, and general well-being. This creates a complicated web of interactions that may confound results and, as is the case with clinical evaluations, requires careful consideration.

## Conclusions

Animal models will never perfectly mimic the human condition, but are intended to guide scientific understanding and provide insight into specific processes involved with post-ischemic recovery. As such, several studies have already proved essential in identifying processes that may underlie the functional improvements resulting from CIMT, and the body of literature continues to grow. The remaining challenge is to ensure that this experimental research is effectively translated in order to capitalize on existing knowledge, identify common findings, and ensure positive collaborations toward improving post-stroke recovery.

## Competing interests

The authors declare that they have no competing interests.

## Authors’ contributions

JLT and AT wrote, edited, and approved the manuscript.
